# Recombinant human ACE2: acing out angiotensin II in ARDS therapy

**DOI:** 10.1186/s13054-017-1882-z

**Published:** 2017-12-13

**Authors:** Haibo Zhang, Andrew Baker

**Affiliations:** 1 Keenan Research Centre for Biomedical Science, St. Michael’s Hospital, Room 619, LKSKI, 30 Bond Street, Toronto, ON M5B 1W8 Canada; 2 Interdepartmental Division of Critical Care Medicine, Departments of Anesthesia and Physiology, University of Toronto, Toronto, ON Canada

**Keywords:** Lung injury, Renin-angiotensin system, Clinical trial

Acute respiratory distress syndrome (ARDS) is a devastating inflammatory lung disorder that is frequently associated with multiple organ dysfunction leading to high mortality. The mechanisms underlying ARDS are multi-factorial, and are thought to include the renin-angiotensin system (RAS) [1, 2].

The RAS is a coordinated complex hormonal cascade that is composed of angiotensinogen, angiotensin-converting enzyme (ACE) and its homolog angiotensin converting enzyme 2 (ACE2), and angiotensin II (Ang II) type 1 and type 2 receptors (AT1, AT2). ACE cleaves the decapeptide Ang I into the octapeptide Ang II, while ACE2 cleaves a single residue from Ang II to generate Ang 1-7, which in turn blocks Ang II and inhibits ACE [3]. Thus, the ACE2 axis negatively regulates the ACE axis.

Great attention has been focused on the role of the RAS in blood pressure homeostasis and cardiovascular function, but there is also increasing interest in understanding the pathophysiological role of the RAS in lung. While only 20% of capillary endothelial cells in all other organs, including the heart, express ACE, it is detectable in the entire capillary network of the alveoli in human lung [4]. Therefore, conversion of Ang I to Ang II can readily occur in the lung by abundant ACE in pulmonary vessels. This may contribute to rapid responses of vasoconstriction in the pulmonary circulation and low blood flow, leading to ventilation/perfusion mismatch in conditions such as tissue hypoxia. On the other hand, ACE2 is primarily produced in Clara cells and type II alveolar epithelial cells [5] and epithelial injury is a critical event in the development of ARDS in humans; thus, the ability to produce ACE2 is severely impaired, resulting in dominant ACE activities during ARDS and/or ventilator-induced lung injury (VILI) [1, 6].

Increasing evidence has emerged that reactive oxygen species (ROS), especially nicotinamide adenine dinucleotide phosphate (NADPH) oxidases and hydrogen peroxide (H_2_O_2_), act as upstream regulators of RAS and ACE activity in various cells and tissues [7]. The RAS in turn induces production of ROS that function as intracellular and intercellular second messengers to modulate many downstream signaling cascades. In normal conditions, the interplay between the ROS and RAS is important in maintaining pulmonary function and integrity. Under ARDS and VILI conditions, this vicious cycle feedback loop contributes to lung injury and remodeling through oxidative damage [6, 8].

Midkine (MK), a heparin-binding growth factor, has been recently demonstrated as a novel modulator of RAS in the context of ARDS and VILI [6]. The plasma concentration of MK increased dramatically in patients with ARDS [6], and an up-regulation of MK in lung epithelial cells is reported in response to cyclic mechanical stress [6]. Exposure to MK protein results in an enhanced ACE expression in primary human lung cells [9]. MK has been shown to stimulate the RAS by acting as an upstream signaling molecule of Ang II and mediates lung–kidney crosstalk leading to development of RAS-associated fibrosis [9].

The RAS—specifically Ang II via AT1 and AT2 receptors—has a number of effects: (1) induction of pulmonary vasoconstriction and vascular permeability in response to hypoxia resulting in pulmonary edema; (2) stimulation of the lung production of inflammatory cytokines directly and indirectly by targeting bradykinin; (3) acceleration of the Fas-induced apoptosis in alveolar epithelial cells; and (4) promotion of extracellular matrix synthesis and human lung fibroproliferation [10]. These effects of the RAS highlight the crucial role of Ang II in ACE/ACE2-regulated ARDS. Indeed, enhanced ACE activity and decreased ACE2 activity contribute to lung injury during cyclic stretch of human lung epithelial cells and to VILI in animal models [1, 6]. In models of ARDS, the use of ACE2 gene knockout mice demonstrated that ACE2 and Ang 1-7 are protective [2].

The use of Ang II receptor blockers or ACE inhibitors has been effective in decreasing lung injury in animal models, but this approach could have potential side effects, including systemic hypotension in humans. Since ACE2 protected the lung from developing ARDS and functioned as a coronavirus receptor for severe acute respiratory syndrome [11], the recombinant ACE2 (rACE2) protein may have an important place in protecting ARDS patients and as a potential therapeutic approach in the management of emerging lung diseases such as avian influenza A infections [12].

Khan et al. [13] recently reported the results of a phase II trial examining the safety and efficacy of using GSK2586881, a recombinant human ACE2 (rhACE2) in patients with ARDS. They showed that administration of a broad range of doses of GSK2586881 were safe without causing significant hemodynamic changes. The use of twice-daily doses of GSK2586881 infusion resulted in a rapid decrease in plasma Ang II levels and increase in Ang 1-7 and Ang 1-5 levels, as well as a trend to a decrease in plasma IL-6 concentrations. This pilot study opens the prospect for further large trials that are powered to assess clinical outcomes.

Considerations for future large trials using rhACE2 in patients with ARDS and VILI include: 1) large variations in plasma Ang II levels may make it difficult to identify responders (identifying those with elevated Ang II concentrations and a higher ratio of ACE2/ACE activity [1] may help); 2) the human ACE gene contains a polymorphism where one particular allele increases ACE and Ang II activities, and the homozygous state correlates with higher mortality in human ARDS [8, 14]—this may provide an opportunity for better risk stratification; and 3) since soluble ACE2 has a short half-life in vivo, a continuous infusion of rhACE2 may improve efficacy [15].

ARDS continues to be a major clinical problem without any effective pharmacologic intervention. Identifying which patients are more likely to benefit from rhACE2 represents an exciting prospect.

## Abbreviations

ACE: Angiotensin-converting enzyme
ACE2: Angiotensin converting enzyme 2
Ang 1-7: Angiotensin 1-7
Ang II: Angiotensin II
ARDS: Acute respiratory distress syndrome
RAS: Renin-angiotensin system
rhACE2: Recombinant human ACE2
SP-D: Surfactant protein D.
